# Post-COVID syndrome or persistent COVID and depression

**DOI:** 10.1192/j.eurpsy.2023.1708

**Published:** 2023-07-19

**Authors:** S. Castelao-Almodovar, R. Ojea Quintana, A. Arrieta Artigas, A. Arce de la Riva, B. Tejero Soriano

**Affiliations:** 1 Psiquiatría; 2 Hospital Universitario Puerta de Hierro Majadahonda, Majadahonda, Spain

## Abstract

**Introduction:**

Since the start of the COVID19 pandemic, numerous patients have exhibited symptoms related to the viral infection once the acute phase has resolved. The most frequent are fatigue or weakness, cognitive difficulties, insomnia, and anxiety or depression. It has been observed that the persistence of these symptoms is more common in cases of severe infections.

**Objectives:**

We expose a case that exemplifies it; A 60-year-old man suffering from severe COVID19 infection during 2021, with bilateral pneumonia and secondary pneumothorax. Three months after the acute episode, he continues to present related symptoms, such as dyspnea, asthenia, arthromyalgia, nausea, hyporexia, memory lapses, anxiety and depressive mood with apathy, anhedonia and suicidal ideas.

**Methods:**

The patient starts follow-up in Mental Health and antidepressant treatment with Vortioxetine 10mg. In the following months he presented significant improvement consisting in decrease of the asthenia, dyspnea, arthromyalgia and especially in anxious symptoms and depressive mood, disappearing the apathy, anhedonia and suicidal ideation. However, the persistence of memory failures draws attention, which remain in a similar degree or with slight subjective improvement.

The exploration and complementary test were the following:

- Chest CT: Hydropneumothorax, parenchymal infiltrates, alveolar consolidations, left lamellar pneumothorax.
- Head CT, complete analysis, microbiological and cytological studies without relevant resolution.
- Assessment by the Rehabilitation and Neurology service.

**Results:**

This case exposes the existence of a post-COVID syndrome, where the symptoms related to the infection persist, including anxious-depressive symptoms of moderate-severe intensity. The different diagnoses that were considered were the following: Post-COVID syndrome, Adjustment disorder with mixed anxious-depressive symptomatology, Depressive episode.

**Conclusions:**

We consider that in this case and in others that are similar, which are increasingly common in routine clinical practice, the etiopathogenesis of the syndrome is of interest. We found that it is difficult to discern the origin of the symptoms, not being able to differentiate an adaptive difficulty to the infection situation (added to the COVID pandemic context), versus a more organic affectation that improves when receiving pharmacological treatment, as in this case with antidepressants.

**Disclosure of Interest:**

None Declared

